# US detection of renal and ureteral calculi in patients with suspected renal colic

**DOI:** 10.1186/2036-7902-5-S1-S3

**Published:** 2013-07-15

**Authors:** Gianfranco Vallone, Giuseppina Napolitano, Paolo Fonio, Gabriele Antinolfi, Antonio Romeo, Luca Macarini, Eugenio Annibale Genovese, Luca Brunese

**Affiliations:** 1 Department of Biomorphological and Functional Sciences, University of Naples Federico II, Naples, Italy; 2 Department of Health Science, University of Molise, Campobasso, Italy; 3 Institute of Diagnostic and Interventional Radiology, University of Turin , Turin, Italy; 4 Department of Radiology, University of Foggia, Foggia, Italy; 5 Department of Radiology, University of Cagliari, Cagliari, Italy

## Abstract

**Purpose:**

The purpose of this study was to determine whether the color Doppler twinkling sign could be considered as an additional diagnostic feature of small renal lithiasis (_5mm).

**Methods:**

181 patients underwent CT scans performed for other pathologies; the images were also analyzed by a radiologists to identify the incidental presence of renal lithiasis equal to or smaller than 5 mm.

These patients underwent an abdominal ultrasound examination, including grayscale analysis of the kidneys and color Doppler. Lithiasis were divided into three groups, on the basis of the diagnostic agreement provided by CT and gray scale results. Then, the twinkling sign sensitivity was assessed in the three groups.

**Results:**

The twinkling sign was positive in 177 out of 206 lithiasis (86 %) visible on CT, while the grayscale was absolutely positive in 98 out of 206 lithiasis (47.6%) and doubtful positive in 71 out of 206 lithiasis (31%).

The twinkling sign was positive in 100% of absolutely positive and doubtful positive lithiasis on bmode, and in 8 out of 31 lithiasis not visible on b-mode.

**Conclusions:**

In the diagnosis of small renal lithiasis, integrating gray-scale with color Doppler may be the most suitable procedure, because the color-Doppler twinkling sign is able to confirm the doubtful diagnosis of renal lithiasis and to detect some lithiasis that are not visible on b-mode.

## Introduction

It is estimated that about 5% of American women and 12% of men will develop a renal lithiasis at some time in their life, and these figures are rising [1].

Medium and large renal lithiasis (> 5mm) can be easily detected with 2D ultrasonography due to the different echogenicity with the adjacent parenchyma and the posterior acoustic shadowing [2].

In the case of small renal lithiasis (_5 mm), differential diagnosis between suspected renal lithiasis and hyperechoic foci caused by other factors (e.g. vascular and/or parenchymal calcifications, clots, arcuate arteries) is difficult [3,4]. The identification of an additional sonographic feature of renal lithiasis is therefore important to reduce the number of false negatives and false positives as well as the number of examinations [5,6]. The Doppler twinkling sign has often been associated with lithiasis [7-9].

The aim of this study was to investigate whether the Doppler twinkling sign is linked to the presence of small renal lithiasis (_5 mm).

## Patients and methods

Between April 2004 and February 2007, 181 consecutive patients with various abdominal pathologies (not directly linked to renal lithiasis) were included in our study (109 males and 72 females; age range 36-65, mean age 52). All patients underwent CT, showing at least one renal lithiasis. All patients then underwent US at our department.

The CT scans, performed for other pathologies with a four-detector spiral CT Philips MX8000, were analyzed by a radiologist to identify the incidental presence of renal lithiasis equal to or smaller than 5 mm [10].

US and color Doppler examinations were performed with GE Logiq 9 (General Electric Company, Milan, Italy), a commercially available real-time US system, equipped with a 1,5- to 4.5-MHz convex transducer (4C General Electric Company, Milan, Italy). Two sonographers performed the examinations. They were aware of the number of renal lithiasis but were blinded to their side and location. A different investigator carried out the data analysis.

Each ultrasound examination included a grey-scale and a color Doppler renal examination.

During the US examination, the physician focused on the detection of renal lithiasis, equal to or smaller than 5mm [10]. In order to visualize any posterior acoustic shadowing, focal zones were placed at the same depth as or slightly deeper than the lithiasis, with careful control of the grey-scale gain setting. A multicolor map was used during the color Doppler sonography; the color window size was adjusted to include the entire lesion and adjacent tissue. The color Doppler gain was set just below the threshold for color noise in order to eliminate the noise of adjacent soft tissues [11-13].

The sonographic appearance of renal lithiasis was analyzed on the basis of size, echogenicity difference between suspected lithiasis and adjacent tissue and posterior acoustic shadowing. The CT scan determined the size of the lithiasis. The echogenicity difference between the lithiasis and the adjacent tissue was recorded as marked, slight or absent. The posterior acoustic shadowing was recorded as present or absent.

Lithiasis were divided into three groups:

1. Lithiasis that, on grey-scale, provided the same diagnosis of CT (i.e., CT positive and absolutely positive on ultrasound (US), same side and location). Lithiasis showing posterior shadow cone and/or marked echogenicity difference (compared to the surrounding parenchyma) were classified as absolutely positive on US.

2. Lithiasis that on grey-scale raised some diagnostic doubts with comparison to CT (i.e., CT positive and doubtful positives on US, same side and location). Lithiasis showing no posterior shadow cone and slight echogenicity difference (compared to the surrounding parenchyma) were classified as doubtful positives on grey-scale.

3. Both B-mode and CT scan demonstrated identical efficacy in identifying lithiasis (i.e., CT positive but not visible on grey-scale images). Lithiasis classified as not visible on grayscale images had the following characteristics: they were observed on CT in a clear area of the kidney, but did not appear on grey-scale images in the same area as hyperechogenic foci with posterior shadow cone or with at least a minimal echogenicity difference.

CT negative grey-scale positive lithiasis (regardless of diagnostic certainty) were excluded from our study.

All 181 patients with lithiasis underwent a color-Doppler sonography to assess the presence and intensity of the twinkling sign.

The color-Doppler, grey-scale and CT results were compared in order to assess the twinkling sign sensitivity in each of the three groups.

## Results

On abdominal CT scans, 81 selected patients showed 206 renal lithiasis equal to or smaller than 5 mm. The diameter of renal lithiasis ranged from 2.6 to 5.0 mm (average diameter 4.1 mm).

Out of 206 CT positive lithiasis, 98 (47.6%) were absolutely positive on gray-scale. Out of the 98 lithiasis, 14 were hyperechogenic foci with posterior shadow cone, but with a slight echogenicity difference, 60 showed both posterior shadow cone and a marked echogenicity difference, and 24 showed a marked echogenicity difference without posterior shadow cone. So, the posterior shadow cone appeared 74 out of 98 times, while marked echogenicity difference was detected 84 times.

All 98 lithiasis of this group were twinkling sign positive (100%).

Out of 206 CT positive lithiasis, 71 (34.5%) were grey-scale doubtful positive with the following characteristics: hyperechogenic foci, slight echogenicity difference and no posterior shadow cone.

All 71 lithiasis of this group were twinkling sign positive (100%).

The remaining 37 (18%) CT positive lithiasis were not visible on grey-scale in the same area they were observed on CT. Out of these 37 lithiasis, 8 were twinkling sign positive.

In total, the twinkling sign was positive in 177 out of 206 lithiasis (86 % sensibility) visible on CT, while the grey-scale was absolutely positive in 98 out of 206 lithiasis (47.6%) and doubtful positive in 71 out of 206 lithiasis (31%).

Results are summarized in Tables 1 and 2.

**Table 1:** Twinkling sign and group of grey-scale examinations

Results of grey-scale examinations	Numbers of calculi	Twinkling sign sensitivity
absolutely positive	*98*	*100% (98/98)*
doubtful positives	*71*	*100% (71/71)*
not visible	*37*	*21,62% (8/37)*

**Table 2:** Presence and Sensitivity of US features

US features	Presence of the feature	Sensitivity%
*B-mode marked echogenicity difference*	*64/206*	*31,06%*
*Posterior acoustic shadowing*	*74/206*	*35,92%*
*Twinkling sign*	*177/206*	*85,92%*
*Combination of posterior acoustic shadowing and bmode marked echogenicity difference (and/or)*	*98/206*	*47,57%*

## Discussion

Medical literature shows that the most valuable echographic parameters for renal lithiasis, i.e. the posterior shadow cone and the hyperechogenic foci with marked echogenicity difference from the surrounding tissue have a low sensitivity compared to the total cases of renal lithiasis. This factor explains why CT is so often required in the diagnosis of suspected renal lithiasis.

**Figure 1:**
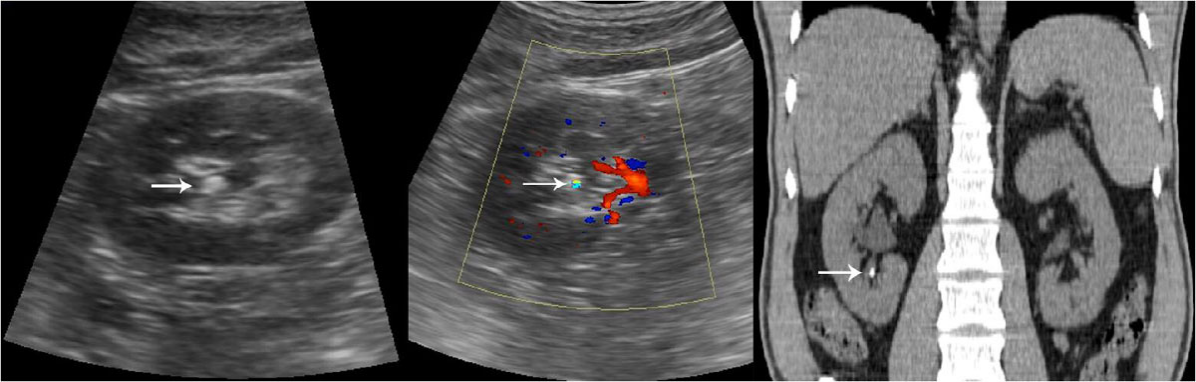
Gray-scale sonogram shows hyperechoic spot with posterior acoustic shadowing. Color Doppler sonogram shows a twinkling sign. A small renal stone. Unenhanced CT confirms the presence of renal stone.

The twinkling sign [14] is generated from the ‘noise’ stemming from rough interfaces composed of sparse reflectors, such as lithiasis or vascular calcifications, which split the sonography beam in a complex unit of waves. This produces a mix of red and blue pixels on color Doppler as turbulent flows [15,16] (Figure 1).

As shown in literature, the twinkling sign can also be associated with vascular and/or parenchymal calcifications [17,18]. It is possible to differentiate between these entities: when the twinkling sign is produced by vascular calcifications, it appears near a structure that at the same time produces a pulsing color signal due to the blood flow [19-22]. When the twinkling sign is produced by intraparenchymal calcifications, it differs from lithiasis due to its parenchymal location [23-25].

This study demonstrates that the twinkling sign is often associated with the presence of small renal lithiasis (_5 mm) and its identification can increase ultrasonography capabilities almost to CT levels. In our study, the twinkling sign was present in 177 (86%) out of 206 renal lithiasis identified with CT.

In the first group of lithiasis (CT positive and absolutely positive to grey-scale ultrasound, same side and location), the twinkling sign was always positive, proving to be as valuable and sensitive a sign as the other grey-scale parameters considered in medical literature. It is very interesting to compare the grey-scale images and the color-Doppler results of the second group of lithiasis (CT positive and doubtful positive on grey-scale images).

In the case of these lithiasis, which are very frequent due to the echostructural complexity of the renal medulla, the grey-scale parameters are not capable of providing a clear diagnosis. CT is required in order to do this. The twinkling sign is 100% positive in the lithiasis of this group, leading the way to a new diagnostic approach. It provides a new diagnostic tool for ultrasound to be used in those cases (71 out of 206 cases in our study, 34.5%) where classical ultrasound semeiotics only provide a doubtful diagnosis.

**Figure 2:**
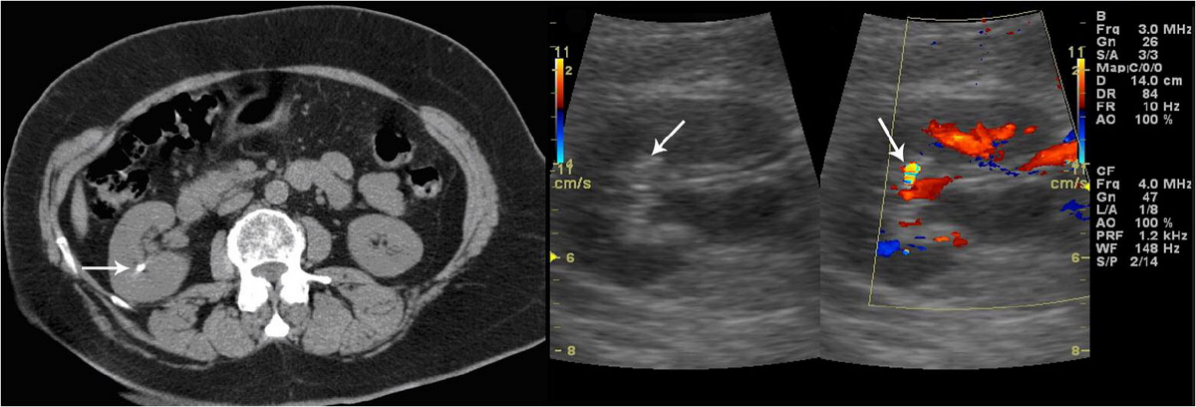
Unenhanced CT confirms the presence of renal stone. Grey-scale sonogram shows small hyperechoic spot without posterior acoustic shadowing. Color Doppler sonogram shows a twinkling sign.

This means that the use of ionizing radiation is not necessary in the case of hyperechogenic foci with slight echogenicity differences and without posterior shadow cones on twinkling sign positive grey-scale images (Figure 2).

**Figure 3:**
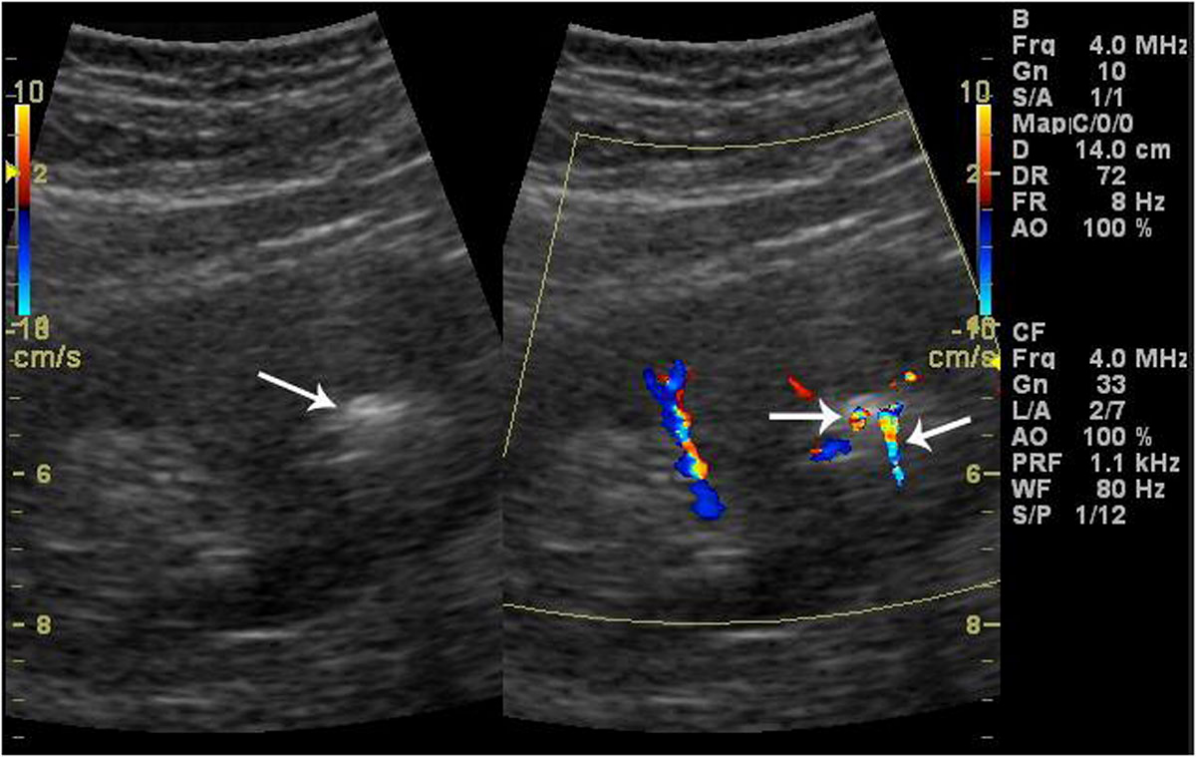
Two small renal stones with calyceal dilatation. Grey-scale sonogram shows only one hyperechoic spot with posterior acoustic shadowing. Color Doppler sonogram reveals two twinkling signs.

As regards the third group of lithiasis (CT positive but not visible on grey-scale ultrasound), the twinkling sign was positive in 8 out of 37 cases (22% sensitivity) (Figure 3). Although this method demonstrates low sensitivity in the detection of this group of lithiasis it is still useful as these lithiasis are not detectable by B-mode. The twinkling sign therefore provides a significant diagnostic advantage compared to grey-scale images, especially if we compare the sensitivity of the two techniques in the diagnosis of lithiasis in this specific group (21.6% vs 0%).

Moreover, the color-Doppler must be accurate and rigorous in order to identify the twinkling sign, aiming at finding a scanning plan in which the ultrasound beam is exactly perpendicular to the lithiasis. Only in this specific case does the lithiasis produce the twinkling sign. Identifying the twinkling sign is therefore much more difficult in color-Doppler because for these lithiasis, there are no grey-scale parameters that might raise some doubts and lead the physician to focus on a certain area of the kidney on color-Doppler. False negatives (29 out of 206) on the twinkling signs could be produced by the interference of obesity, intestinal meteorism and lack of cooperation by the patient with the ultrasound technique.

It may thus be inferred that, for a clear diagnosis, the twinkling sign is a much more sensitive parameter (86%) on the total number of lithiasis compared to the posterior shadow cone (36%) and to the marked echogenicity difference (31%), and also compared to both parameters (47.6%) (Tab.2).

As already mentioned, CT negative, grey-scale positive lithiasis (regardless of diagnostic certainty) were excluded from our study. Lithiasis might be CT negative because of the presence of false positives on grey-scale and, quite seldom, because of sampling mistakes due to CT parameters that were not compatible with the dimensions of the small lithiasis. In our opinion, including this group of lithiasis would imply a loss of objectivity in our study, leading our team to make philosophical speculations on the existence or non-existence of what might appear on the images. For this reason, we decided to focus on clearly diagnosed lithiasis, using CT as selection technique.

## Conclusions

In conclusion, the twinkling sign showed the highest sensitivity in the case of certain or doubtful diagnosis of renal lithiasis on grey-scale. In some cases, the twinkling sign was also able to show the presence of lithiasis when grey-scale images were unable even to assume their presence.

We believe that in cases of suspected small renal lithiasis, integrating grey-scale images with color Doppler is the most appropriate procedure in the diagnosis of renal small lithiasis.

## Competing interests

The authors declare that they have no competing interests.

## Declarations

This article has been published as part of *Critical Ultrasound Journal* Volume 5 Supplement 1, 2013: Topics in emergency abdominal ultrasonography. The full contents of the supplement are available online at http://www.criticalultrasoundjournal.com/supplements/5/S1. Publication of this supplement has been funded by the University of Molise, University of Siena, University of Cagliari, University of Ferrara and University of Turin.
